# Congenital Anomalies of the Female Genital Tract: A Comprehensive Review

**DOI:** 10.7759/cureus.56753

**Published:** 2024-03-23

**Authors:** Swati M Dahiphale, Jyotsana Potdar, Neema Acharya, Garapati Jyotsna, Saloni, Rahul Desale

**Affiliations:** 1 Obstetrics and Gynaecology, Jawaharlal Nehru Medical College, Datta Meghe Institute of Higher Education & Research, Wardha, IND; 2 Radiodiagnosis, Jawaharlal Nehru Medical College, Datta Meghe Institute of Higher Education & Research, Wardha, IND

**Keywords:** future directions in research, management strategies, psychosocial impact, diagnosis techniques, asrm müllerian anomalies classification, female genital tract anomalies

## Abstract

This comprehensive review provides an in-depth examination of congenital anomalies of the female genital tract, explicitly focusing on the American Society for Reproductive Medicine (ASRM) Müllerian Anomalies Classification. The classification system is crucial for standardizing communication and guiding accurate diagnoses in clinical practice. The review explores the diverse clinical presentations, etiological factors, and diagnostic modalities associated with these anomalies. Management strategies, ranging from conservative approaches to advanced reproductive technologies, are discussed in the context of individualized treatment plans based on the ASRM classification. The psychosocial impact of female genital tract anomalies is thoroughly examined, emphasizing the importance of holistic care and patient-centered approaches. Looking toward the future, the review outlines emerging research areas, including advances in diagnosis techniques, innovative treatment modalities, and genetic studies. It ultimately underscores the need for a comprehensive understanding of physical and psychosocial dimensions, offering insights for healthcare professionals to navigate this complex landscape and improve the lives of affected individuals.

## Introduction and background

Congenital anomalies of the female genital tract encompass various structural irregularities that arise during embryonic development. These anomalies may involve the uterus, cervix, fallopian tubes, and vagina, manifesting as deviating from the typical anatomical configuration. The exploration of these congenital anomalies is crucial for clinicians, researchers, and healthcare professionals to comprehend the intricate nature of female reproductive health [1-2].

Understanding female genital tract anomalies is paramount in reproductive medicine, gynecology, and obstetrics. These anomalies can profoundly impact reproductive outcomes, influencing fertility, pregnancy, and childbirth [3-5]. These structural irregularities may also contribute to a spectrum of gynecological issues, affecting menstrual health, urinary and bowel function, and overall quality of life for affected individuals. Recognizing the clinical implications of these anomalies is essential for providing optimal patient care and guiding therapeutic interventions [6].

This comprehensive review aims to delve into the intricate landscape of congenital anomalies of the female genital tract, thoroughly examining their classification, epidemiology, etiology, clinical presentation, diagnosis, and management. The primary objectives of this review are to synthesize existing knowledge, highlight recent advancements in the field, and provide clinicians and researchers with a comprehensive resource for understanding and navigating the complexities associated with female genital tract anomalies. By addressing these objectives, we strive to contribute to the ongoing discourse on reproductive health, thereby fostering informed decision-making in clinical practice and guiding future avenues of research.

## Review

Classification of congenital anomalies

American Society for Reproductive Medicine (ASRM) Müllerian Anomalies Classification

**Figure 1:**
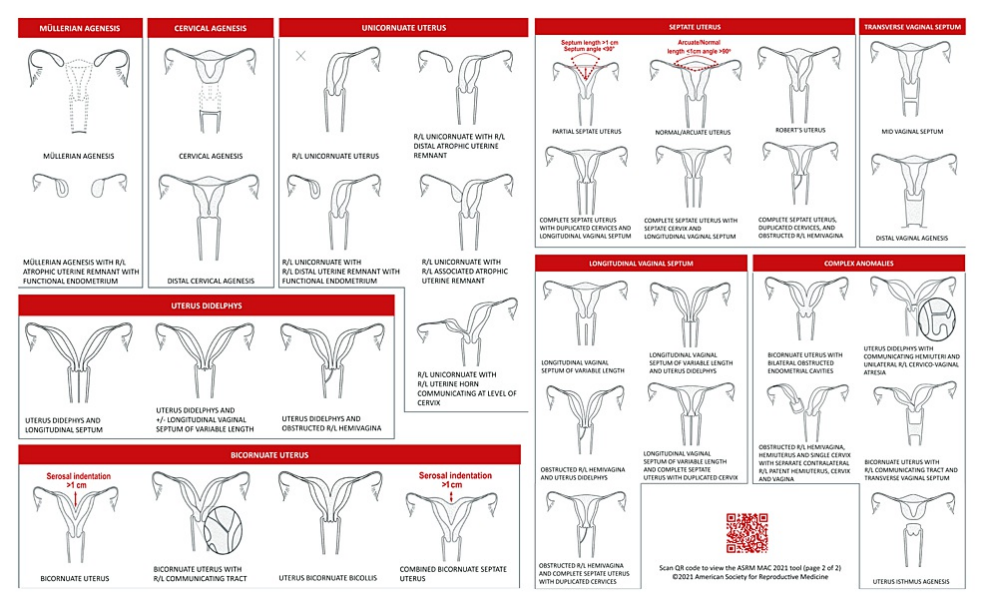
ASRM Müllerian Anomalies Classification 2021 Open access journal under a CC-BY license. Contributed by the American Society for Reproductive Medicine [3]

The American Society for Reproductive Medicine (ASRM) has developed a comprehensive classification system for Müllerian anomalies, providing a standardized framework for categorizing variations in the female genital tract. This classification system aids in clinical diagnosis, treatment planning, and communication among healthcare professionals [3,7]. The following are the main classes within the ASRM Müllerian Anomalies Classification 2021 (Figure 1).

Epidemiology

Prevalence of Female Genital Tract Anomalies

The prevalence of female genital tract anomalies is estimated to be around 4-6.9% [8]. However, specific anomalies may have different prevalence rates. For example, the imperforate hymen has an epidemiology of 1:1000-2000 in female individuals, while Müllerian agenesis has an epidemiology of 1/5000 in female individuals [9]. These anomalies are being diagnosed more frequently due to advances in imaging techniques, and they are present in about 7% of the general population, with an even higher prevalence in the infertile population [10].

Age and Ethnicity Patterns

The occurrence of female genital tract anomalies often exhibits age and ethnicity patterns. Some anomalies may become more apparent or symptomatic during certain life stages, such as adolescence, when individuals may experience menstrual irregularities or difficulties with sexual intercourse. The age at which women seek reproductive assistance or become pregnant may also influence the identification of these anomalies [11]. Moreover, variations in the prevalence of female genital tract anomalies among different ethnic groups have been documented. Genetic factors, including variations in the expression of specific genes related to reproductive organ development, may contribute to these ethnic disparities. Further research is necessary to elucidate the genetic and environmental factors that underlie the observed variations in prevalence among diverse populations [12].

Association With Other Medical Conditions

Female genital tract anomalies may be associated with other medical conditions, either congenital or acquired. For instance, anomalies such as uterine septum or bicornuate uterus may coexist with renal anomalies in a condition known as the Mayer-Rokitansky-Küster-Hauser (MRKH) syndrome. Understanding these associations is crucial for comprehensive patient care and may impact treatment strategies [13]. Additionally, specific genetic syndromes, such as Turner syndrome, may be associated with gonadal dysgenesis and contribute to the overall spectrum of female genital tract anomalies. Exploring these associations is essential for providing holistic care and identifying potential underlying genetic or syndromic etiologies [7].

Etiology and pathogenesis

Genetic Factors

Hox genes: The Hox gene family, recognized for its pivotal role in determining body segment identity during embryonic development, is crucial in forming the female reproductive organs. These genes contribute to the precise patterning and differentiation of tissues, including the Müllerian ducts, which give rise to the uterus and other reproductive structures. Disruptions in the expression or function of Hox genes have been implicated in various congenital anomalies, particularly uterine malformations. Understanding the intricate regulatory role of Hox genes is fundamental to unraveling the molecular basis of reproductive organ development and associated anomalies [14].

WNT signaling pathway: The WNT signaling pathway, a key player in embryonic development, is significant in Müllerian duct formation. This pathway governs critical cellular processes, influencing cell fate determination and tissue differentiation. Aberrations in the WNT signaling pathway have been linked to uterine anomalies, emphasizing the importance of precise genetic regulation in developing female reproductive structures. Investigating the molecular intricacies of WNT signaling provides valuable insights into the complex cascade of events that shape the embryonic Müllerian ducts and contribute to the formation of the uterus [15].

Homeobox A10 (HoxA10) and homeobox A11 (HoxA11): HoxA10 and HoxA11 are specific members of the Hox gene family that play a central role in uterine organogenesis. These genes regulate cell differentiation, proliferation, and tissue patterning during the development of the uterus. Mutations or altered expression levels of HoxA10 and HoxA11 have been associated with uterine anomalies, underscoring the critical role of these specific regulatory genes in normal uterine development. Elucidating the precise molecular mechanisms controlled by HoxA10 and HoxA11 contributes to our understanding of the genetic basis of uterine malformations. It provides potential targets for therapeutic interventions to correct these anomalies at the molecular level [16].

Environmental Factors

Maternal smoking and drug exposure: Maternal smoking and exposure to certain drugs during pregnancy, certain SSRIs like Fluvoxamine, Lexapro, Paxil, Prozac, Symbyax, and Zoloft have emerged as significant factors associated with an elevated risk of congenital anomalies, including those affecting the female genital tract. The impact of these substances on embryonic development can disrupt the intricate processes of organ formation, potentially leading to structural abnormalities in reproductive organs. The vulnerability of the developing fetus to the teratogenic effects of smoking and certain drugs underscores the importance of public health initiatives aimed at reducing maternal exposure to these risk factors. Understanding the specific mechanisms by which these substances influence female genital tract development is crucial for designing preventive strategies and promoting maternal and fetal well-being [17].

Maternal diabetes: Maternal diabetes, particularly when present during the critical period of organogenesis, has been identified as a significant risk factor for congenital anomalies, including those affecting the female genital tract. The developing female reproductive organs may be particularly susceptible to the effects of maternal hyperglycemia, which can disrupt normal developmental processes. Glycemic control during pregnancy becomes paramount in mitigating the risk of congenital anomalies. Close monitoring and management of maternal diabetes aim to provide optimal conditions for fetal development and reduce the potential impact on the female genital tract. Insights into the specific pathways through which maternal diabetes influences organogenesis can inform targeted interventions and enhance prenatal care strategies to minimize the risk of congenital anomalies in affected individuals [18].

Hormonal Influences

Anti-Müllerian hormone (AMH): AMH, primarily produced by the developing testes in males, is a critical regulator in the regression of the Müllerian ducts during male fetal development. In females, the absence or altered regulation of AMH may contribute to the persistence of Müllerian structures, leading to congenital anomalies in the female genital tract. Anomalies such as uterine malformations or vaginal septa may result from incomplete regression or abnormal development of the Müllerian ducts. Investigating the nuanced interplay between AMH and the female reproductive tract during embryogenesis provides valuable insights into the molecular mechanisms underlying these anomalies, potentially informing targeted therapeutic strategies or interventions [19].

Estrogen and progesterone: Balanced levels of estrogen and progesterone are paramount for the normal development of the female reproductive organs. During critical periods of embryogenesis, hormonal imbalances can disrupt the intricate processes involved in forming the female genital tract. Deviations from the optimal hormonal milieu may lead to structural abnormalities affecting the uterus, cervix, or other reproductive structures. Understanding the specific effects of estrogen and progesterone on cellular differentiation, tissue patterning, and organogenesis is essential for unraveling the complex etiology of congenital anomalies in the female genital tract. Insights into hormonal influences contribute to developing targeted interventions to restore hormonal balance and potentially prevent or correct anomalies during the early stages of fetal development [20].

Teratogenic Agents

Thalidomide and diethylstilbestrol (DES): Historical instances involving thalidomide and DES serve as poignant reminders of the teratogenic potential associated with certain medications. Thalidomide, prescribed in the late 1950s and early 1960s for morning sickness, resulted in severe limb anomalies and other congenital disabilities when taken during pregnancy. Similarly, DES, a synthetic estrogen prescribed to prevent miscarriages between the 1940s and 1970s, was later linked to reproductive tract anomalies and an increased risk of clear cell adenocarcinoma in female offspring. These examples underscore the critical importance of vigilance and thorough assessment of drug safety during pregnancy. Insights gained from these historical incidents have contributed to enhanced regulations and awareness, emphasizing the need for rigorous testing and cautious prescribing practices to safeguard fetal development and prevent congenital anomalies [21].

Clinical presentation

Asymptomatic Cases

A notable characteristic of congenital anomalies of the female genital tract is that a significant proportion of affected individuals may remain asymptomatic. In these cases, the anomalies are often incidentally discovered during routine pelvic examinations, imaging studies, or investigations for unrelated health concerns. Asymptomatic individuals may not experience noticeable disruptions in reproductive or gynecological function, and the anomalies may only become apparent when seeking medical attention for unrelated reasons [2].

Reproductive Issues

Infertility: Female genital tract anomalies exert a significant impact on fertility, influencing the intricate processes of conception and implantation. Structural variations within the uterus and cervix pose challenges to successful conception, with uterine anomalies like septate or bicornuate uterus altering the spatial dynamics of the uterine cavity. These variations can impede the optimal conditions required for the implantation of a fertilized egg, thereby affecting the chances of achieving a viable pregnancy. Anomalies such as the unicornuate uterus and uterine didelphys are also associated with an increased risk of infertility, emphasizing the importance of a thorough understanding of the specific anatomical challenges each anomaly presents. Managing infertility in the context of female genital tract anomalies requires a tailored approach that addresses the underlying structural factors influencing reproductive success [1].

Recurrent pregnancy loss: Certain congenital anomalies elevate the risk of recurrent pregnancy loss, creating additional hurdles for individuals aspiring to build a family. Anomalies like septate uterus or bicornuate uterus can disrupt the regular vascular supply to the uterine lining, contributing to recurrent miscarriages. Addressing these structural abnormalities becomes paramount in the management of recurrent pregnancy loss, as interventions aimed at correcting uterine anomalies can enhance the likelihood of a successful pregnancy. Understanding the intricate relationship between female genital tract anomalies and recurrent pregnancy loss informs clinical decision-making, guiding healthcare providers in implementing targeted interventions to optimize reproductive outcomes for individuals facing these challenges [22].

Menstrual Disorders

Congenital anomalies of the female genital tract can influence menstrual patterns and contribute to menstrual disorders. Women with septate uterus or other structural anomalies may experience abnormal uterine bleeding, dysmenorrhea, or irregular menstrual cycles. The presence of a transverse vaginal septum can lead to obstructive symptoms, causing difficulties in the standard passage of menstrual flow [23].

Urinary Tract and Bowel Symptoms

Urinary symptoms: Female genital tract anomalies, particularly those involving the uterus, can manifest in urinary symptoms due to their anatomical proximity to the bladder. Anomalies such as a septate uterus or other uterine abnormalities may exert pressure on the adjacent bladder, resulting in urinary symptoms such as increased frequency, urgency, or incontinence. The altered anatomy can disrupt the normal spatial relationship between the uterus and bladder, leading to functional disturbances. In more complex cases, such as cloacal anomalies involving the fusion of the urinary and reproductive tracts, shared channels may result in intricate urinary manifestations. Understanding the interplay between female genital tract anomalies and urinary symptoms is crucial for comprehensive patient care and may guide appropriate interventions to alleviate these symptoms [24].

Bowel symptoms: Female genital tract anomalies can contribute to bowel symptoms, mainly when anomalies affect the rectovaginal septum or involve complex conditions such as cloacal anomalies. Anomalies affecting the rectovaginal septum may lead to symptoms such as constipation or difficulties with bowel movements. The presence of a transverse vaginal septum, for example, can cause obstructive symptoms that impact bowel function [25]. Cloacal anomalies, characterized by the fusion of the rectum, vagina, and urinary tract, may result in shared channels and complex interactions affecting bowel function. A comprehensive understanding of these anatomical relationships is essential for healthcare providers to address bowel symptoms effectively and improve the overall quality of life for individuals with female genital tract anomalies [25].

Diagnosis

Medical History and Physical Examination

Medical history: Initiating the diagnostic process for congenital anomalies of the female genital tract begins with a comprehensive medical history. This essential step involves gathering pertinent information such as menstrual history, reproductive experiences, any instances of infertility or recurrent pregnancy loss, and details regarding urinary or bowel symptoms. Uncovering the patient's medical background provides valuable insights into potential underlying causes and aids in formulating a practical diagnostic approach. Furthermore, thoroughly examining family history is crucial to identifying genetic factors contributing to these anomalies, allowing for a more holistic understanding of the patient's condition [26].

Physical examination: A detailed physical examination, including a meticulous pelvic examination, is indispensable in diagnosing congenital anomalies of the female genital tract. This examination serves to assess both the external and internal genital anatomy. Specific physical findings, such as the presence of a transverse vaginal septum, abnormal cervical configuration, or the identification of a vaginal dimple, provide valuable clues pointing toward particular anomalies. The examination may also extend to assess for associated anomalies in other organ systems, offering a comprehensive understanding of the patient's overall health. The insights gained from the physical examination contribute to developing a targeted diagnostic plan, guiding subsequent imaging and laboratory investigations for a precise and tailored diagnosis [27].

Imaging Techniques

Ultrasound: Ultrasonography, particularly transvaginal ultrasound, is a fundamental imaging modality in evaluating female genital tract anomalies. This non-invasive technique enables high-resolution visualization of the uterus, cervix, and ovaries, offering valuable insights into their structural integrity. While less detailed, transabdominal ultrasound can complement the assessment in some instances. Ultrasound is particularly adept at detecting uterine anomalies, including septate or bicornuate uterus, and contributes to evaluating ovarian function. This imaging modality is an initial and often essential step in the diagnostic process, guiding subsequent investigations and informing treatment decisions [28].

MRI: MRI emerges as a powerful and versatile tool for characterizing the intricate anatomy of the female reproductive organs. With its exceptional soft tissue contrast, MRI is particularly valuable in delineating complex anomalies such as uterine didelphys or septate uterus. It provides detailed information about the extent of structural variations, aiding in treatment planning, especially before surgical interventions. MRI's ability to capture three-dimensional images enhances diagnostic precision, making it a cornerstone in the diagnostic armamentarium for female genital tract anomalies [29].

Hysterosalpingography (HSG): HSG involves the injection of contrast material into the uterine cavity and fallopian tubes, followed by X-ray imaging. This procedure is instrumental in assessing the morphology of the uterus and fallopian tubes. HSG can reveal abnormalities such as a septate uterus or blockages in the fallopian tubes, providing crucial information for both diagnostic and therapeutic purposes. It is commonly employed as part of the infertility workup, offering insights into potential factors contributing to reproductive challenges and guiding subsequent management strategies. The dynamic nature of HSG allows for real-time observation of contrast flow, enhancing its utility in assessing tubal patency and uterine cavity abnormalities [30].

Laboratory Tests

Hormonal assays: Hormonal assays, encompassing assessments of estrogen, progesterone, and anti-Müllerian hormone (AMH) levels, play a pivotal role in gaining insights into the hormonal milieu and ovarian function. These tests contribute to the comprehensive evaluation of the endocrine status, offering valuable information about the regulatory mechanisms governing the female reproductive system. Hormonal imbalances detected through these assays may be associated with specific congenital anomalies of the female genital tract and can influence reproductive outcomes. By providing a snapshot of the hormonal environment, these assays aid healthcare providers in tailoring treatment strategies and addressing potential endocrine-related factors contributing to reproductive challenges [31].

Genetic testing: Genetic testing assumes significance, particularly in cases where there is suspicion of underlying genetic syndromes or when multiple family members are affected by female genital tract anomalies. Various genetic testing modalities may be employed, including karyotyping, fluorescence in situ hybridization (FISH), or molecular genetic testing to identify specific gene mutations associated with these anomalies. The results of genetic testing not only contribute to a precise diagnosis but also have implications for family planning and genetic counseling. Understanding the genetic basis of female genital tract anomalies enables healthcare providers to offer more personalized and targeted care, focusing on addressing the underlying genetic factors that may contribute to these conditions [32].

Management and treatment

Conservative Approaches

Observation: In instances where female genital tract anomalies are asymptomatic or do not significantly impact reproductive function, a conservative approach involving observation may be deemed appropriate. This approach recognizes that not all anomalies require active intervention, and careful monitoring through regular imaging and clinical assessments allows healthcare providers to track any changes over time. Observation is a valuable strategy for cases where the anomaly is stable, not causing discomfort or functional impairment, and where the potential benefits of intervention do not outweigh the risks. This approach aligns with the principle of providing individualized care that considers each patient's unique circumstances and preferences [2].

Hormonal therapy: Hormonal therapy emerges as a viable option in cases where hormonal imbalances are identified or anomalies are associated with specific hormonal dysregulation. This therapeutic approach aims to regulate menstrual cycles, alleviate symptoms, and optimize reproductive outcomes. For instance, in the context of uterine anomalies affecting endometrial receptivity, hormonal therapy may be employed to create a more conducive environment for implantation. The targeted use of hormones, such as estrogen or progesterone, can address specific challenges related to the hormonal milieu, contributing to improved reproductive function. Hormonal therapy is often tailored to the patient's needs, with close monitoring to assess its efficacy and make adjustments as necessary. This approach exemplifies a proactive, patient-centered strategy for managing female genital tract anomalies [33].

Surgical Interventions

Hysteroscopic septum resection: Hysteroscopic septum resection represents a minimally invasive surgical procedure designed to correct anomalies such as a septate uterus or other uterine septa. During this procedure, a hysteroscope is introduced through the cervix into the uterine cavity, providing direct visualization for the surgeon. The septum is excised using specialized instruments, facilitating the restoration of a more normal uterine configuration. Hysteroscopic septum resection is particularly valuable for improving reproductive outcomes by creating an environment conducive to implantation. This minimally invasive approach often results in shorter recovery times and reduced postoperative discomfort compared to traditional surgical interventions [34].

Vaginoplasty: Vaginoplasty is a surgical intervention designed to address anomalies such as vaginal agenesis or transverse vaginal septum. This procedure involves the creation or reconstruction of the vaginal canal, aiming to enhance sexual function and enable the passage of menstrual flow. The choice of specific vaginoplasty techniques depends on the anomaly's nature and individual patient circumstances. Vaginoplasty plays a crucial role in improving the quality of life for individuals with congenital anomalies affecting the vagina, providing both functional and cosmetic benefits [35].

Uterine anomaly repair: Surgical correction of uterine anomalies, such as bicornuate or unicornuate uterus, may be considered in cases where the anomaly is associated with recurrent pregnancy loss or infertility. These procedures involve reconfiguring the uterine shape to optimize reproductive function. The choice of a specific surgical approach depends on factors such as the type and severity of the uterine anomaly. Uterine anomaly repair is geared towards improving the likelihood of successful pregnancies by addressing structural abnormalities that may hinder proper implantation and fetal development. This surgical intervention is an integral component of a comprehensive treatment plan for individuals seeking to overcome reproductive challenges associated with uterine anomalies [36].

Assisted Reproductive Technologies (ART)

In vitro fertilization (IVF): assisted reproductive technologies (ART), notably IVF, offer a valuable option for individuals with specific female genital tract anomalies. IVF involves the fertilization of an egg with sperm outside the body, followed by the transfer of the resulting embryo into the uterus. This method is a powerful tool to overcome anatomical challenges within the female reproductive tract, providing a pathway to successful pregnancy. By bypassing certain structural obstacles, such as uterine anomalies, IVF enhances the chances of successful fertilization, implantation, and, ultimately, a healthy pregnancy. This approach is particularly beneficial for individuals facing infertility related to anatomical variations within the female genital tract [26].

Surrogacy: Surrogacy emerges as a consideration in cases where uterine anomalies significantly impact the ability to carry a pregnancy to term. This alternative involves another woman, the surrogate, carrying the pregnancy on behalf of the intended parents. Surrogacy provides a viable solution for individuals with uterine factors that may affect gestation, offering the opportunity to experience parenthood despite challenges related to the female reproductive tract. The collaborative nature of surrogacy involves careful legal and ethical considerations to ensure the well-being of all parties involved. This option serves as an avenue for individuals and couples to realize their dream of building a family when traditional pregnancy may be compromised due to structural anomalies in the uterus [37].

ASRM Müllerian anomalies classification and clinical Implications

Significance of ASRM Classification

ASRM Müllerian Anomalies Classification 2021 holds significant clinical implications. This classification system aims to standardize terminology, facilitate communication, and simplify searches in scientific databases. ASRM Müllerian Anomalies Classification is a valuable framework for clinicians, researchers, and healthcare professionals in diagnosing and managing female genital tract anomalies. Its significance lies in providing a standardized system for consistent communication, accurate diagnosis, and improved understanding of the various anomalies. The ASRM classification categorizes anomalies based on anatomical features, identifying specific types and guiding appropriate interventions [38].

Standardized communication: The ASRM classification system promotes clear, standardized communication within the healthcare community. By providing a structured framework for describing female genital tract anomalies, this classification facilitates seamless interdisciplinary collaboration. Healthcare providers across various specialties can use a common language to convey information about specific anomalies, ensuring that accurate and pertinent details are conveyed consistently. This standardized communication enhances the efficiency of healthcare delivery, promoting effective coordination among professionals involved in the diagnosis, treatment, and management of female genital tract anomalies [39].

Accurate diagnosis: The ASRM classification system is a valuable tool for accurate diagnoses of specific female genital tract anomalies. Through this system, healthcare providers can precisely identify the type and severity of an anomaly, allowing for a more nuanced understanding of the patient's condition. Accurate diagnosis is paramount in tailoring personalized treatment plans that address each individual's unique needs and challenges. The ASRM classification not only aids in identifying the primary anomaly but also assists in recognizing any associated features or complications. This precision in diagnosis contributes to informed decision-making and facilitates proactive interventions, ultimately improving the overall quality of care for individuals with female genital tract anomalies [40].

Impact on Treatment Planning

Individualized treatment approaches: The ASRM classification system provides a foundation for developing more individualized and targeted treatment approaches for female genital tract anomalies. By categorizing anomalies into distinct classes, healthcare providers can tailor interventions to address the specific challenges associated with each type of anomaly. This individualized approach recognizes the unique circumstances of each patient, considering factors such as the type and severity of the anomaly, the patient's reproductive goals, and any coexisting medical conditions. Tailoring treatment plans based on the ASRM classification enhances the precision and effectiveness of interventions, increasing the likelihood of successful outcomes and optimizing the overall quality of care for individuals with female genital tract anomalies [40].

Optimizing reproductive function: Treatment planning guided by the ASRM classification is designed to optimize reproductive function for individuals with female genital tract anomalies. For example, surgical correction of uterine anomalies, informed by the specific classification, may enhance the chances of successful conception and a healthy pregnancy. This approach recognizes that the impact of anomalies on reproductive function varies widely, and interventions must be tailored accordingly. Individualized treatment plans can be particularly crucial in cases where anomalies coexist or where additional factors, such as hormonal imbalances, contribute to reproductive challenges. By optimizing reproductive function through targeted interventions, healthcare providers strive to improve the overall reproductive outcomes and enhance the well-being of individuals affected by female genital tract anomalies [41].

Considerations for Reproductive Outcomes

Reproductive counseling: The ASRM classification system plays a vital role in guiding reproductive counseling for individuals with female genital tract anomalies. By categorizing anomalies into distinct classes, healthcare providers can effectively communicate the potential impact of specific anomalies on fertility and pregnancy outcomes. This counseling is crucial for supporting individuals in making informed decisions about their reproductive journey. Through clear and standardized communication facilitated by the ASRM classification, healthcare providers can discuss the unique challenges associated with each anomaly class, address concerns, and guide available options. Reproductive counseling enables individuals to make decisions aligned with their goals and values, empowering them to navigate the complexities of family planning with a comprehensive understanding of their situation [36].

Risk assessment for complications: The ASRM classification system enables healthcare providers to conduct a more nuanced risk assessment for complications associated with female genital tract anomalies. Different classes of anomalies carry varying risks, including the potential for recurrent pregnancy loss or preterm birth. By categorizing anomalies and understanding their implications, healthcare providers can assess these risks more accurately. This knowledge empowers providers to implement preventive measures or interventions tailored to the individual's situation, optimizing reproductive outcomes and minimizing the likelihood of complications. The ability to assess risks associated with specific classes of anomalies enhances the precision of medical guidance and contributes to a comprehensive and personalized approach to reproductive care [39]. 

Psychosocial impact

Quality of Life Issues

Body image and self-esteem: Individuals with visible genital tract anomalies often contend with challenges related to body image and self-esteem. Societal norms and expectations regarding physical appearance can exacerbate these challenges, leading to feelings of self-consciousness and impacting overall self-perception. The visibility of genital anomalies may evoke societal judgments or comparisons, contributing to a sense of being different or outside conventional beauty standards. This can have profound psychosocial implications, potentially influencing relationships, confidence, and the overall well-being of individuals. Cultivating a positive body image and self-esteem is crucial, and support from healthcare professionals and mental health services can play a pivotal role in addressing these challenges [42].

Sexual function and intimacy: Anomalies affecting the reproductive organs can have a significant impact on sexual function and intimacy. Pain or discomfort during sexual intercourse may be associated with specific anomalies, affecting the overall quality of the sexual experience. The challenges these anomalies pose may influence intimacy's emotional and physical aspects. Open communication between individuals and their healthcare providers is essential to address concerns related to sexual function and intimacy. Tailored interventions, including medical treatments or counseling, can contribute to improving sexual well-being and fostering healthy intimate relationships [43].

Social stigma and disclosure: Stigmatization and societal misconceptions surrounding female genital tract anomalies may contribute to feelings of isolation. Whether to disclose one's condition to others becomes a complex consideration, balancing the desire for understanding and support with concerns about potential judgment. Social stigma may arise from cultural taboos or limited awareness about these conditions, further complicating the disclosure process. Addressing societal misconceptions through education and advocacy efforts can contribute to reducing stigma creating a more supportive environment for individuals with genital tract anomalies. Mental health support, including counseling or support groups, can provide a safe space for individuals to navigate these challenges [44].

Fertility-related stress: For individuals desiring children, fertility-related stress becomes a significant aspect of the psychosocial impact. The challenges associated with conception, potential fertility treatments, and the risk of adverse reproductive outcomes may contribute to heightened stress levels and emotional strain. The uncertainty surrounding fertility can evoke feelings of frustration, sadness, or anxiety. Comprehensive support, including fertility counseling, mental health services, and educational resources, can help individuals cope with fertility-related stress. Addressing the psychosocial impact of fertility challenges is integral to holistic care, promoting emotional well-being alongside medical interventions [44].

Psychological and Emotional Aspects

Anxiety and depression: Coping with the diagnosis and management of female genital tract anomalies can significantly increase levels of anxiety and depression. The emotional impact stems from the uncertainty surrounding reproductive outcomes, the potential need for medical interventions, and societal pressures related to body image and fertility. Individuals may experience heightened stress as they navigate the complexities of their condition. Healthcare providers play a crucial role in recognizing and addressing the psychological aspects of these challenges, offering emotional support, and connecting individuals with appropriate mental health resources [45].

Impact on relationships: The psychosocial impact of female genital tract anomalies extends to interpersonal relationships, including those with partners, family members, and friends. Open communication about the challenges associated with these conditions is crucial for maintaining supportive relationships and addressing any relational strain. Partners may also experience their emotional responses to the diagnosis, and fostering understanding and empathy is essential. Supportive environments that encourage dialogue and shared decision-making contribute to the emotional well-being of individuals and their loved ones [46].

Reproductive grief: Individuals facing difficulties with fertility or experiencing recurrent pregnancy loss may grapple with reproductive grief. The emotional toll of unsuccessful attempts at conception and the loss of pregnancies can lead to profound feelings of sadness, frustration, and grief. Acknowledging and validating these emotions is essential for providing compassionate care. Healthcare providers can play a role in supporting individuals through the grieving process, offering resources for emotional support, and guiding them toward appropriate mental health services [47].

Coping mechanisms and support: Developing effective coping mechanisms and accessing emotional support are integral components of managing the psychological and emotional aspects of female genital tract anomalies. Support from healthcare providers, mental health professionals, and support groups can provide individuals with the tools to navigate the emotional challenges associated with their condition. Encouraging healthy coping strategies, such as mindfulness, counseling, or peer support, empowers individuals to build resilience and address the impact on their mental well-being. A multidisciplinary approach that integrates medical care with psychosocial support enhances the overall quality of care for individuals facing female genital tract anomalies [48].

Future directions and research

Advances in Diagnosis Techniques

3D imaging and virtual reality: Future developments in the field may witness the integration of advanced imaging technologies, such as three-dimensional (3D) ultrasound and virtual reality (VR), offering a more detailed and immersive visualization of female genital tract anatomy. These technologies can potentially revolutionize the diagnostic process, providing healthcare professionals with enhanced insights into the structural nuances of anomalies. 3D imaging and VR can contribute to improved accuracy in diagnosis and offer surgeons valuable information for preoperative planning. This technological integration may redefine the standards of care by providing a more comprehensive and interactive understanding of female genital tract anomalies [49].

Artificial intelligence (AI) in imaging analysis: Incorporating AI into imaging analysis is poised to play a significant role in diagnosing female genital tract anomalies. AI algorithms can efficiently process and analyze imaging data, assisting in identifying and classifying anomalies with high accuracy. By automating certain aspects of image interpretation, AI has the potential to improve diagnostic efficiency, reduce interpretation errors, and enhance overall precision. Integrating AI-based tools into the diagnostic workflow holds promise for optimizing the use of imaging modalities in assessing female genital tract anomalies [50].

Functional imaging techniques: Future advancements may explore the application of functional imaging techniques, such as functional MRI (fMRI), to gain insights into the physiological aspects of the female reproductive organs. Beyond structural considerations, functional imaging can provide information about the dynamic functionality of the reproductive system. Understanding the functional implications of anomalies can contribute to a more comprehensive approach to diagnosis and treatment planning. Functional imaging techniques have the potential to elucidate how anomalies impact physiological processes, guiding healthcare providers in tailoring interventions that address both structural and functional aspects of female genital tract anomalies [51].

Emerging Treatment Modalities

Regenerative medicine and tissue engineering: Advances in regenerative medicine and tissue engineering represent a promising frontier for developing innovative treatment modalities for female genital tract anomalies. These strategies involve leveraging the principles of regenerative biology to stimulate the regeneration or reconstruction of damaged or absent tissue in the female genital tract. This holds particular promise for conditions such as vaginal agenesis or structural deficiencies, where regenerating functional tissue is essential. Regenerative medicine approaches may offer novel therapeutic options that restore normal anatomy and function, potentially transforming the treatment landscape for certain female genital tract anomalies [52].

Gene therapy and molecular interventions: Molecular and gene therapy may emerge as a frontier for addressing genetic factors contributing to female genital tract anomalies. Targeted approaches could involve modifying gene expression or correcting genetic mutations associated with these anomalies. By intervening at the molecular level, gene therapy offers the potential for more precise and personalized treatments. Understanding the genetic underpinnings of specific anomalies may open avenues for therapeutic interventions that address the root causes, potentially transforming the landscape of treatment for individuals with genetic predispositions to female genital tract anomalies [53].

Non-invasive therapies: Exploring non-invasive therapies, such as focused ultrasound or other targeted energy modalities, represents a novel approach to correcting certain female genital tract anomalies. These non-invasive interventions aim to provide alternatives to traditional surgical procedures, potentially reducing invasiveness and optimizing patient outcomes. Non-invasive therapies may offer advantages such as shorter recovery times, minimized risk of complications, and improved patient experience. Investigating and developing non-invasive modalities for the correction of female genital tract anomalies reflects a commitment to advancing patient-centric care and expanding the array of available treatment options [54].

Genetic and Molecular Research

Genetic profiling and precision medicine: Advances in genetic profiling techniques can potentially revolutionize our understanding of the genetic underpinnings of female genital tract anomalies. Genetic profiling allows for a more comprehensive analysis of an individual's genetic makeup, uncovering specific variations that may contribute to anomalies. This knowledge forms the basis for precision medicine, where treatment strategies can be tailored to the unique genetic profile of each patient. Precision medicine in the context of female genital tract anomalies involves personalized interventions that consider the individual's genetic factors, paving the way for more targeted and effective treatments [55].

Epigenetic studies: Exploring epigenetic factors influencing the development of female genital tract anomalies adds a layer of complexity to our understanding of these conditions. Epigenetic studies investigate how environmental influences interact with genetic factors through mechanisms that regulate gene expression. Unraveling the epigenetic landscape may provide insights into the etiology of female genital tract anomalies and identify potential therapeutic targets. Understanding the interplay between genetics and epigenetics enhances our ability to comprehend the multifaceted nature of these anomalies. It may open avenues for interventions that address both genetic and environmental factors [56].

Biomarkers for reproductive outcomes: Ongoing research focuses on identifying biomarkers associated with reproductive outcomes in individuals with female genital tract anomalies. Biomarkers are measurable indicators that can provide valuable information about the likelihood of successful conception, guide treatment decisions, and assess the impact of interventions on fertility. The discovery of reliable biomarkers holds the potential to refine prognostic assessments, allowing healthcare providers to offer more accurate predictions regarding reproductive outcomes. This area of the research aligns to enhance predictive capabilities and tailor interventions to optimize fertility and reproductive success in individuals with female genital tract anomalies [57].

## Conclusions

This comprehensive review explored the intricate realm of congenital anomalies of the female genital tract, shedding light on key findings and implications for clinical practice. The ASRM Müllerian Anomalies Classification has emerged as a pivotal tool, providing a standardized framework for communication and precise diagnosis. Our exploration of clinical presentations, etiological factors, diagnostic modalities, and management strategies underscores the need for a holistic approach that considers physical and psychosocial aspects. As we navigate the complexities of female genital tract anomalies, it is evident that future directions in research hold tremendous potential. Advances in diagnosis techniques, emerging treatment modalities, and ongoing genetic and molecular investigations offer hope for improved precision in diagnostics, expanded therapeutic options, and the growth and development of personalized medicine in this field. As healthcare professionals strive to integrate these advancements into practice, a patient-centered approach will be paramount, guided by informed decision-making and clear communication. Moreover, continued research into advanced imaging technologies, regenerative medicine, and genetic studies will further contribute to understanding and managing these anomalies, ultimately enhancing the quality of care and outcomes for affected individuals.
